# Improved Measurements of the Physical Properties of Oriental Lacquers Using Atomic Force Microscopy and a Nanoindenter

**DOI:** 10.3390/polym13091395

**Published:** 2021-04-26

**Authors:** Hye Hyun Yu, Jung-Ah Lim, Kang-Bong Lee, Yeonhee Lee

**Affiliations:** 1 Advanced Analysis Center, Korea Institute of Science and Technology, Seoul 02792, Korea; 091615@kist.re.kr; 2 Center for Opto-Electronic Materials and Devices, Korea Institute of Science and Technology, Seoul 02792, Korea; jalim@kist.re.kr; 3 National Agenda Research Division, Korea Institute of Science and Technology, Seoul 02792, Korea; leekb@kist.re.kr

**Keywords:** Oriental lacquer, natural polymer, physical properties, atomic force microscopy, nanoindenter

## Abstract

Researchers have widely investigated Oriental lacquers to identify the chemical composition and have elucidated corresponding polymerization mechanisms using rigorous analytical techniques. However, researchers generally test the physical properties of Oriental lacquers by conventional methods that are perhaps overly simplistic. Here, we propose accurate and quantitative methods for evaluating the physical properties of Korean, Vietnamese, and Myanmarese lacquer films using atomic force microscopy (AFM), a nanoindenter, and a 90° peel tester. We obtained surface images of the lacquers in accordance with drying time using scanning electron microscopy and AFM. The Korean lacquer film exhibited fast hardening speed, enhanced hardness, and strong adhesion strength compared with the other lacquers, although the Myanmarese lacquer film had a smoother surface than the Korean lacquer film. We used our characterization approach for evaluating a mixed Korean/Myanmarese (50/50 *w*/*w*) lacquer. Our proposed measurement techniques for Oriental lacquer films provided results that agreed with qualitative results from conventional tests. Force–distance curves in AFM and force–displacement with nanoindenter for Oriental lacquer films showed more accurate and quantitative data on the mechanical properties. Thus, researchers will find our approach useful when they optimize the chemical compositions and improve the physical properties of Oriental lacquer films for industrial applications.

## 1. Introduction

Oriental lacquer is an eco-friendly natural polymer that has been handled for thousands of years in Asian countries. One collects lacquer sap by tapping *Anacardiaceae* family lacquer trees and drying the sap into a durable, beautiful, and brilliant film for naturally occurring coatings [1,2,3,4,5]. Over the course of drying, only water evaporates; organic solvents are not involved. Oriental lacquer is common in modern arts and handicrafts. Researchers also use such lacquer as a protective, adhesive layer for functional materials because it has excellent characteristics and performance, such as waterproofing, high strength, anti-bacterial properties, and durability [6,7,8,9,10,11].

Traditional lacquer sap is generally produced from the following trees: *Toxicodendron vernicifluum* (Japan, Korea, and China), *Toxicodendron succedaneum* (Taiwan and Vietnam), and *Gluta usitata* (Thailand and Myanmar). The sap collected from a lacquer tree is milky-yellow and a water-in-oil emulsion [1,2,12]. Lacquer sap is composed of 60–70% lipids (such as urushiol, laccol, or thitsiol), 5–10% plant gum (polysaccharides and enzymes), 3–5% glycoproteins, and 20–30% water. Researchers commonly use various analytical techniques to obtain information on the chemical components of Oriental lacquers [13,14,15,16,17]. The main constituents of natural lacquer—lipids—are catechol derivatives with a linear alkenyl group: a C15 or C17 chain. The drying mechanism of Oriental lacquer mainly proceeds by two processes [18,19,20,21,22]. The first step is laccase-catalyzed oxidation. The catechol lipids in lacquer react with laccase to generate semiquinone radicals. Radical transfer produces catechol dimers, trimers, oligomers, and polymers through nuclear–nuclear (C–C) coupling. The second step is auto-oxidation, which proceeds when the concentration of the catechol monomer decreases to 30% [2,23,24]. The double bonds in the triene side chains auto-oxidize to form allyl radicals that participate in the crosslinking reaction with radicals in other side chains. Both laccase-mediated oxidation and auto-oxidation continue to harden the lacquer film.

Recently, many research groups have elucidated lacquer chemistry by using rigorous analytical techniques to understand fundamental phenomena and improve lacquer’s advantageous properties for industrial applications. Atomic force microscopy (AFM) has been frequently used to investigate the surface topographies of lacquer films, especially refined Oriental lacquer with light sensitizers, urushi with lignin in bio-composite films, and superhydrophobic composite coating of nitrocellulose lacquer to improve lacquer’s physical properties [25,26,27,28]. Surface properties of Oriental lacquer have been controlled and applied in a lot of practical applications, for example, fibrous porous media with roughened surfaces, such as surface topology [29,30]. Oxidative polymerization of lipids produces a cross-linked network structure that is related to the durability of lacquer film coatings. The physical properties of Asian lacquer films, such as hardness, adhesion, surface roughness, and drying speed, depend on the type and quantity of catechol lipids. In previous studies, researchers used conventional methods, such as a drying time recorder, viscosity, pencil hardness, and cross-cut tests to measure the physical properties of lacquer films [23,24,31]. For example, the Miyakoshi group characterized and compared the drying speed, UV resistance, viscosity, and rigid-body pendulum physical properties of various Asian lacquers and blended lacquers [32,33]. Although informative, these techniques are perhaps too simplistic for quantitative evaluations.

AFM and nanoindenter are based on the quantitative measurement of mechanical properties and are frequently used to measure various coating materials [34,35]. However, researchers rarely reported quantitative determinations and comparisons of the physical properties of Oriental lacquer films by using rigorous analytical techniques to correlate with simple traditional methods. Results of a drying time recorder, pencil hardness, and cross-cut tests can provide relative comparison and approximate status of lacquer films. However, it is not possible to get more specific results and quantitative values to evaluate the physical properties of the lacquer films. We evaluated Oriental lacquer films in accordance with catechol lipid type and drying time using AFM and a nanoindenter. For measurement of the surface topography and surface drying, the force–distance curve method of AFM was used. A nanoindenter was also used to quantify the hardness properties of lacquer films using force–displacement curve. We compared these results with those obtained by conventional testing methods, such as a drying time recorder, the pencil hardness test, and the cross-cut test. We thus evaluated the physical properties of Oriental lacquer films quantitatively and provided a new pathway for developing and improving the physical properties of natural lacquer films.

## 2. Materials and Methods

### 2.1. Materials

Raw lacquer saps were collected from lacquer trees of *T. vernicifluum* and *G. usitata* grown in Korea and Myanmar, respectively. They were supplied by the local market, Pyung Hwa Shell (Seoul, Korea). The Vietnamese *T. succedaneum* lacquer was obtained by the Vietnam–Korea Institute of Science and Technology (Hanoi, Vietnam). Conventional methods were used to filter pure lacquer saps with Korean traditional paper. Turpentine oil—the solvent—was obtained from a local supplier (PyungHwa Shell, Seoul, Korea). All other solvents were used directly without further purification.

### 2.2. Preperation of Lacquer Films

The blended lacquer was prepared by mixing *G. usitata* with *T. vernicifluum* lacquer sap uniformly with a blending ratio of 1:1 (*w/w*). Turpentine oil was used to make a film-type sample for the physical measurements. The concentration of the lacquers was 50/50 (*v*/*v*) using turpentine oil. After all the lacquer/turpentine oil samples were well-mixed using a vortex mixer, the samples were applied using a blade onto a glass slide that was cleaned with methanol and acetone. The coated lacquer films were dried in a humidity-controlled chamber with 85% relative humidity (RH) at 25 °C. The thickness of the lacquer films was measured with a profilometer (P-10 Tencor, KLA Corporation, Milpitas, CA, USA) and was approximately 5–6 μm thick depending on the coating time.

### 2.3. Instrumentation

Lacquer films prepared on glass slides were used for measuring all the physical properties except for adhesion, for which the lacquer saps were coated onto a stainless-steel plate because of stainless steel’s stronger adhesion compared with glass. The drying time of the lacquer saps was obtained with an automatic AB-3600 drying time recorder (TQC sheen, South Holland, Netherlands). Drying occurred over three stages: dust-free dry (DF), touch-free dry (TF), and hardened dry (HD) [36]. The hardness of the lacquer films was measured with a pencil hardness tester (221-D, Kipae E&T Co., Suwon, Korea) using various types of pencils. The pencil grades were classified as 9H (hardest) to 6B (softest), expressing the hardness degree of a pencil that could break the lacquer film. The adhesion forces were tested using a cross-cut adhesion tester (YCC-230/1, Yoshimitsu, Japan) [37]. The adhesion of the lacquer film on the glass substrate was measured using the 90° peel test (KOPC-B, Kyung-Jin Hitech; Gunpo-si, South Korea). Sample sizes, tape widths, and tape types were determined for optimum conditions after several experiments.

**Figure 1:**
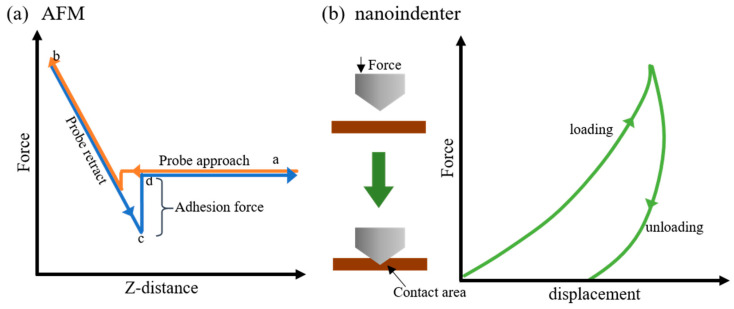
Schematic illustration of (**a**) force–distance curve of AFM and (**b**) force–displacement curve of nanoindenter.

The sample roughness was measured on the lacquer film surface by AFM (XE-100, Park Systems, Suwon, Korea). AFM measurements were conducted with an SD-R30-NCH-10M cantilever (Nanosensors, Neuchâtel, Switzerland) in non-contact mode. Image areas of (40 × 40) μm^2^ were collected at various sample positions. The root-mean-square roughness (*R_rms_*) and average roughness (*R_a_*) were obtained by XEI v.4.3.0 software (Park Systems, Suwon, Korea). Figure 1 shows force–distance curve in AFM and force–displacement with the nanoindenter. In the force–distance curve in AFM, the force is the reaction force that a cantilever takes by pressing on the sample surface, and Z-distance is the deflecting distance of a cantilever. Force–distance curve points were evaluated on the lacquer films where the drying process was ongoing, using a constant force of 2 μN. Field-emission scanning electron microscopy (FESEM) measurements were performed at a voltage of 10 kV with an Inspect F50, FEI (Hillsboro, OR, USA) to investigate the lacquer film surface.

The nanoindentation measurements of the lacquer films were conducted with a nanoindenter (TI-950 Triboindenter, Bruker, Billerica, USA) with a Berkovich diamond tip. While the indenter moves vertically at a constant speed, the indenter displacement and the normal force were measured as shown in Figure 1b. A maximum load of 2 mN was used with constant loading and unloading ratios of 0.1 mN/s to obtain the nanoindentation properties. A maximum load for 2 s was maintained for the nanoindenter. The Oliver–Pharr method was used to calculate the nanoindentation modulus and hardness [38].

## 3. Results and Discussion

### 3.1. Surface Images

**Figure 2:**
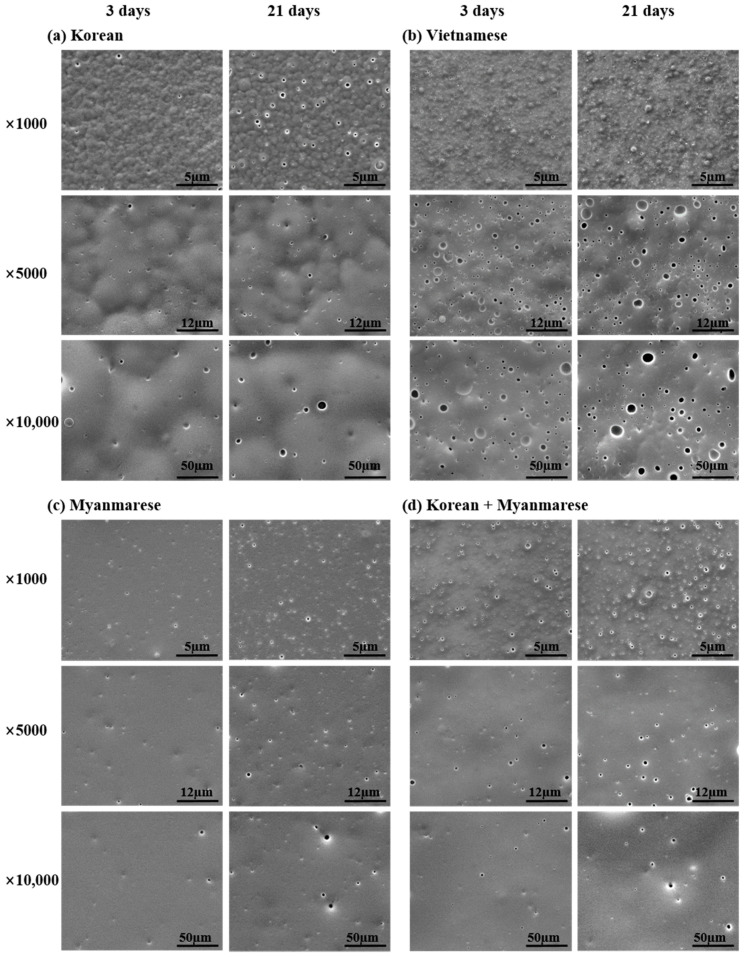
FESEM images of Oriental lacquers as a function of drying time: (**a**) Korean, (**b**) Vietnamese, (**c**) Myanmarese, (**d**) Korean/Myanmarese mixture (1/1, *w*/*w*).

We obtained FESEM images of lacquer film surfaces from various lacquer tree saps at 1000×, 5000×, and 10,000× magnification. Figure 2a–d shows FESEM images of Korean, Vietnamese, Myanmarese, and mixed (Korean/Myanmarese, 1/1 *w*/*w*) lacquer films in accordance with the drying time (3 and 21 d) at room temperature with 85% RH.

Lacquer sap is a water-in-oil emulsion. When lacquer sap is dried to produce a film, the polysaccharides and glycoproteins merge into the lipid component of the emulsion [18,21]. Water evaporates, resulting in micro-cavities in the lacquer film. The size and number of micro-cavities in the lacquer films generally increased after 21 d of drying time. The Vietnamese lacquer film showed more and larger cavities (pits or holes) compared with other lacquer films because the Vietnamese lacquer sap contained comparatively more water (~30% water content). The upper surface layer of the Korean and Vietnamese lacquer films shrank and deformed by enzymatic polymerization over the course of drying. However, the Myanmarese lacquer film had a smooth surface compared with the other lacquer films. The mixed Korean/Myanmarese lacquer film showed a fairly smooth surface compared with the pure Korean lacquer film.

**Figure 3:**
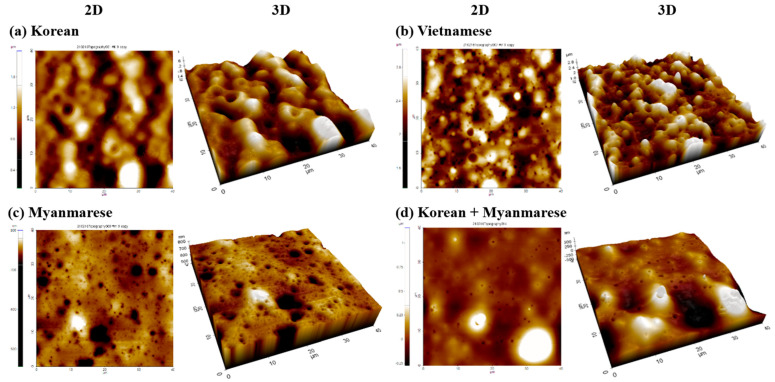
Two-dimensional and three-dimensional AFM images of Oriental lacquers after a drying time of 28 d: (**a**) Korean, (**b**) Vietnamese, (**c**) Myanmarese, (**d**) Korean/Myanmarese mixture (1/1, *w*/*w*).

We also used AFM to investigate the surface topographies in accordance with the various types of lacquer trees. Figure 3a–d show AFM 2D and 3D images of the lacquer films after a 28 d drying time. The topographic images indicated differences in the morphologies of the lacquer films. The surface of the Korean lacquer film was curved. The Vietnamese and Myanmarese lacquer films were rough and relatively flat, respectively. Over the course of drying, the AFM topographies of the lacquer films also showed more holes on the surface that were generated by water evaporation, as shown in the FESEM images. The AFM results provided the *R_rms_* and *R_a_* values of the lacquer films (Table 1). The Korean and Vietnamese lacquers had higher *R_rms_* and *R_a_* values, and the Myanmarese lacquer had comparatively small values of *R_rms_* and *R_a_*. The roughness values of the Korean and Japanese lacquers were similar to each other because they came from the same kinds of *T. vernicifluum* lacquer trees. The surface roughness of the mixed Korean/Myanmarese lacquer film was smoother than the Korean film and rougher than the Myanmarese film.

### 3.2. Traditional Methods for Physical Properties 

To get the simple and useful information on the various lacquer surfaces, drying speed, adhesion property, and hardness were measured using conventional methods, such as drying time recorder, pencil hardness and cross-cut tests. We used an automatic drying time recorder to obtain the drying time of lacquer saps in accordance with three stages [36]: DF, TF, and HD (Table 2). Lacquer drying involves laccase-mediated polymerization of the lipid catechol ring, followed by auto-oxidation of the linear chains. Drying necessitates an appropriate temperature and humidity. We dried the lacquer saps at a temperature of 20–25 °C and an RH of 70–85%. The Korean lacquer had a faster drying time than the other lacquer saps. The HD is the time until the marks of the needle in the recorder disappear completely on the film surface. The lacquer HD times were as follows: Korean, 16 h; Vietnamese, 48 h; and Myanmarese, 14 d. The Korean lacquer had more laccase and catechol lipids with triene-rich content, and thus had a fast drying time. The mixed Korean/Myanmarese lacquer dried within 20 h, a faster drying time than the Myanmarese lacquer.

We evaluated physical properties, such as the pencil hardness of the lacquer films, in accordance with the drying time (Table 3). The Korean lacquer film exhibited a hardness of 3H after 3 d, whereas the Vietnamese and Myanmarese lacquer films exhibited a hardness of 2B and 4B, respectively, after 3 d. The Vietnamese lacquer sap dried after 21 d to 4H hardness, whereas the Myanmarese lacquer sap dried after 21 d to reach 2B hardness. The Korean/Myanmarese mixed lacquer had an improved hardness compared with the Myanmarese lacquer: hardness B after 3 d and consistently 3H after 28 d.

The cross-cut tests for the lacquer films were performed to evaluate the adhesive ability in accordance with ASTM D3359 [38] (Table 4). The cross-cut results showed that most small squares of the Korean lacquer film on a glass slide detached, and we classified the adhesion as 1B (an area of 35–65% was peeled off). Additionally, the Vietnamese and Myanmarese lacquer films on a glass slide exhibited a weaker adhesion corresponding to 0B (an area greater than 65% was peeled off). It was not possible to discriminate the adhesion properties of Oriental lacquers on a glass slide because the adhesion strength between lacquer and glass was too weak to measure it by cross-cut tester. Thus, the lacquer saps were coated onto a stainless-steel plate because of stainless steel’s stronger adhesion compared with glass. The lacquer adhesion on a stainless-steel plate was classified as follows: Korean, 3B; Myanmarese 0B; and mixture, 1B after 3 d. However, the adhesion of lacquer films with the substrate was not changed significantly in accordance with drying time. Physical properties such as the drying time, hardness, and adhesion for the Korean/Myanmarese lacquer mixtures showed improved values compared with those of pure Myanmarese lacquers. One can improve the quality of Myanmarese lacquer by blending it with *T. vernicifluum* lacquer, such as Korean, Japanese, and Chinese lacquers. The drying time, pencil hardness, and cross-cut tests are common straightforward techniques due to their low cost, and they are still accepted in the coating industry. However, they do not provide quantitative values and are limited for the determination of very similar physical properties of lacquers.

### 3.3. Accurate Measurements of Mechanical Properties

After measuring the adhesion properties of the lacquer films using the cross-cut test, we determined the adhesion properties more quantitatively using a 90° peel test (Table 5). We peeled off the lacquer films on a glass slide with adhesion strengths as follows: Korean, 9.41 N/m; Vietnamese, 5.19 N/m; and Myanmarese, 6.96 N/m. We also performed the 90° peel tests as a function of drying time. The peel test results showed that the adhesion strength of the Korean lacquer films decreased as follows: from a value of 9.41 N/m on day 28 to a value of 8.62 N/m on day 60. The 90° peel test results showed the similar trend as the cross-cut test yet provided a quantitative measurement for the adhesion strength of the lacquer films.

**Figure 4:**
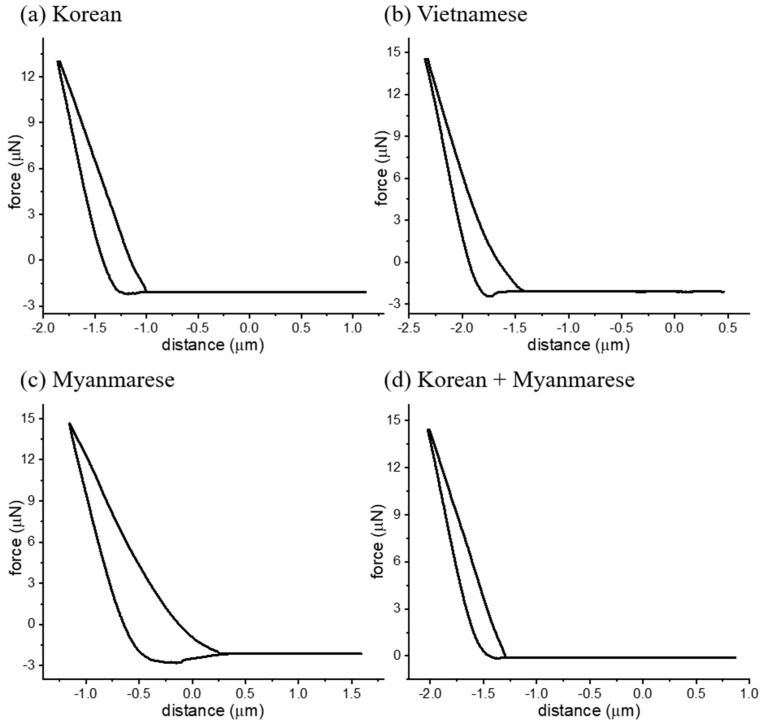
Force–distance curve measurements by AFM for Oriental lacquer films after a drying time of 14 d.

To measure the mechanical properties of the lacquer films more quantitatively and accurately, we used advanced analytical techniques, such as AFM and nanoindentation. We obtained a force–distance curve of the lacquer films to evaluate the differences in the surface adhesion in accordance with the drying time. Figure 4 shows the force–distance curves after 14 d. The Korean lacquer resulted in a force–distance curve of similar shape but with smaller adhesion energy compared with the Vietnamese and Myanmarese lacquers. The Korean/Myanmarese lacquer mixture resulted in a surface that had a similar adhesion energy compared with the Korean lacquer. We also measured the force–distance curves of the lacquers as a function of the drying time (Table 6). We correlated the adhesion energy and Young’s modulus of the lacquer film surfaces with the drying time measurements. The well-dried surface of the lacquer films showed low adhesion energies and high Young’s moduli.

**Figure 5:**
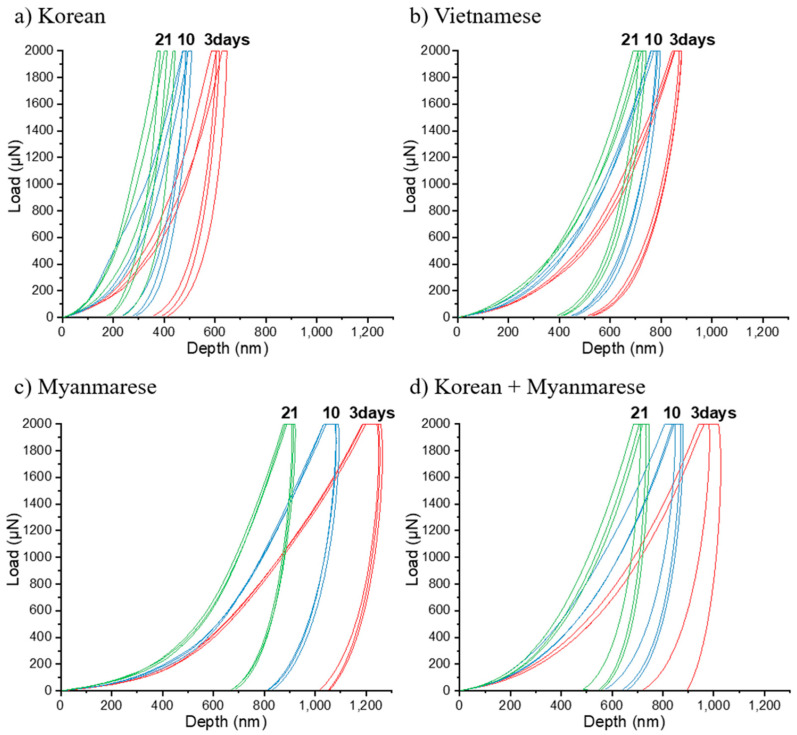
Force–displacement curve measurements with a nanoindenter for Oriental lacquer films in accordance with drying time.

We investigated the nanoindentation properties of the lacquer films with a nanoindenter. Figure 5 shows five force–displacement curves in accordance with drying time. Force–displacement is measured as the indenter tip is pressed into the lacquer film surface with a loading and unloading profile. The nanoindentation result of the lacquer films showed the increasing slope of the loading/unloading curves as a function of the drying time. The large increment of the slopes was observed in force–displacement curves for Myanmarese lacquer film depending on the drying time. We also observed very consistent force–displacement curves for five measurements. The concurrence of force–displacement curves could be due to the effect of the small surface roughness of the Myanmarese lacquer samples. The mechanical properties of the Korean lacquer film were superior to the other lacquers.

**Figure 6:**
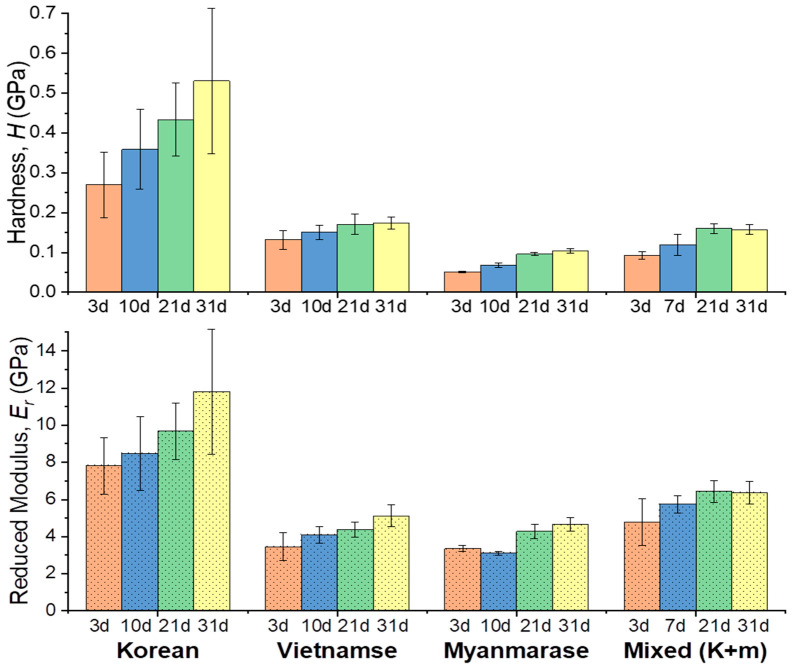
Calculated hardnesses *H* and reduced moduli *E_r_* of Oriental lacquers in force–displacement curves: Korean, Vietnamese, Myanmarese, Korean/Myanmarese mixture (1/1, *w*/*w*).

We calculated the hardness values and reduced moduli of the lacquers as a function of drying time from the nanoindentation results. Figure 6 shows the averaged values of *H* and *Er* of the lacquer films after five measurements at various positions. We also calculated the averaged values of *H* and *Er* for the lacquer films as a function of drying time. After a drying time of 21 d, the Korean lacquer film hardness *H* was 0.431 GPa; this film had a harder surface compared with the other lacquers. The mixed Korean/Myanmarese lacquer film exhibited a hardness *H* of 0.16 GPa and an induced modulus *Er* of 6.43 GPa, which was measured with the decrease in the contact depth (730 nm) compared with the Myanmarese lacquer film (*H*, 0.10 GPa; and *Er*, 4.28 GPa). The values of *H* and *Er* of the lacquer films after 31 d were almost 2× higher than those of the lacquer films after 3 d. Therefore, mechanical properties of Oriental lacquer films can be well expressed quantitatively using force—distance in AFM and force—displacement with a nanoindenter. 

## 4. Conclusions

We investigated the physical properties of various Oriental lacquers—Korean *T. vernicifluum*, Vietnamese *T. succedaneum*, and Myanmarese *G. usitata*—using a drying time recorder, the cross-cut method, and a pencil hardness tester. The Korean lacquer film exhibited a fast drying time, strong adhesion, and hard pencil hardness compared with the Vietnamese and Myanmarese lacquer films. To improve the physical properties of the Myanmarese lacquer, we blended it with the Korean lacquer and evaluated the resulting physical properties. The Korean/Myanmarese lacquer film mixture (1/1, *w*/*w*) showed improved physical properties compared with the pure Myanmarese lacquer film. The drying time, pencil hardness, and cross-cut tests are simple and convenient methods; however, they are not able to discriminate lacquer coating films that have very similar physical properties. Thus, we used complementary or alternative techniques, such as AFM and nanoindenter.

We obtained surface images of the lacquers and their mixtures and compared them using FESEM and AFM. To obtain improved understanding and more accurate measurements, we applied complementary advanced surface techniques. We quantitatively measured the surface hardening state, hardness, and adhesion strength of the Oriental lacquers using force–distance curves in AFM, force–displacement with the nanoindenter, and a 90° peel tester. Although conventional methods provide useful information, more advanced mechanical studies provide accurate and quantitative information toward practical use of Oriental lacquer films. In future work, we will blend *T. vernicifluum* lacquer with inexpensive lacquer or cashew nutshell liquid at different ratios, and we will conduct sensitive characterizations with the proposed methods to obtain exact physical properties of blends that are suitable for industrial applications. Force–distance curves in AFM and force–displacement with the nanoindenter will discriminate mechanical properties of the similar blended lacquers quantitatively and be used complementarily with the traditional methods. Moreover, for material characterization, it is important to understand the interrelationship between the macro-properties measured with simple traditional tests and nano-properties measured with AFM and a nanoindenter.

## Figures and Tables

**Table 1:** Atomic force microscopic evaluation of root-mean-square roughness (*R_rms_*) and average roughness (*R_a_*) of Oriental lacquer films.

Sample (25 °C, 85% RH)	Drying Time (Days)							
	3		7		21		28	
	*R_rms_* (nm)	*R_a_* (nm)	*R_rms_* (nm)	*R_a_* (nm)	*R_rms_* (nm)	*R_a_* (nm)	*R_rms_* (nm)	*R_a_* (nm)
Korean (K)	267.08	211.80	247.83	201.25	242.29	188.11	224.26	180.17
Vietnamese	331.37	262.31	254.14	206.00	197.46	155.52	262.84	200.86
Myanmarese (M)	13.12	7.51	16.10	9.24	17.32	9.98	17.40	10.56
Mixed (K + M) ^1^	104.49	77.96	62.74	46.80	122.97	77.79	112.08	87.32

^1^ The mixed lacquer was a 1/1 (*w*/*w*) mixture.

**Table 2:** Drying time of Oriental lacquer tree saps.

Sample (25 °C, 85% RH)	Drying Time											
	3 h	5 h	8 h	10 h	16 h	20 h	24 h	36 h	48 h	3 d	7 d	14 d
Korean (K)	DF *	DF	DF	TF	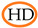	HD	HD	HD	HD	HD	HD	HD
Vietnamese	DF	DF	DF	DF	DF	DF	DF	TF	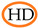	HD	HD	HD
Myanmarese (M)						DF	DF	DF	DF	DF	TF	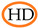
Mixed (K + M)			DF	DF	TF	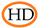	HD	HD	HD	HD	HD	HD

* DF: dust-free dry, TF: touch-free dry, HD: hardened dry.

**Table 3:** Pencil hardness of Oriental lacquer films.

Sample (25 °C, 85% RH)	Drying Time (Days)			
	3	7	21	28
	Pencil hardness			
Korean (K)	3H	3H	4H	3H
Vietnamese	2B	F	4H	4H
Myanmarese (M)	4B	B	2B	4B
Mixed (K + M)	B	3H	H	3H

**Table 4:** Cross-cut test of Oriental lacquer films on a glass slide and a stainless-steel plate.

Sample (25 °C, 85% RH)	Drying Time (Days)		
	3	7	10
	Cross-cut test/glass slide		
Korean (K)	1B	0B	0B
Myanmarese (M)	0B	0B	0B
Mixed (K + M)	0B	0B	0B
	Cross-cut test/stainless steel		
Korean (K)	3B	4B	4B
Myanmarese (M)	0B	0B	0B
Mixed (K + M)	1B	2B	2B

**Table 5:** Adhesion strengths of Oriental lacquer films on a glass slide by 90° peel test.

Sample (25 °C, 85% RH)	Drying Time (Days)	
	28	60
	Adhesion energy (N/m)	
Korean (K)	9.41	8.62
Vietnamese	5.19	4.70
Myanmarese (M)	6.96	2.74

**Table 6:** Force–distance curve measurements by AFM for Oriental lacquer films as a function of drying time.

Sample (25 °C, 85% RH)	Drying Time (Days)		
	3	7	21
	Adhesion energy (J × 10^15^) (±SD)		
Korean (K)	14.7 (±6.9)	8.2 (±3.6)	5.2 (±1.1)
Vietnamese	45.2 (±19.9)	36.1 (±16.7)	12.8 (±5.9)
Myanmarese (M)	1662.1 (±225.4)	531.8 (±73.6)	17.0 (±5.8)
Mixed (K + M)	29.9 (±8.0)	24.3 (±7.3)	1.9 (±0.4)
	Young’s modulus (GPa) (±SD)		
Korean (K)	236.6 (±66.8)	348.1 (±42.6)	496.2 (±60.6)
Vietnamese	141.6 (±38.2)	187.9 (±43.4)	336.2 (±55.3)
Myanmarese (M)	16.3 (±1.0)	27.0 (±3.0)	283.0 (±56.7)
Mixed (K + M)	198.7 (±28.8)	175.9 (±39.0)	679.3 (±79.5)

## Data Availability

Data are contained within the article.

## References

[1] Lu R., Miyakoshi T. (2015). Lacquer Chemistry and Applications.

[2] Lu R., Yoshida T., Miyakoshi T. (2013). Oriental lacquer: A natural polymer. Polym. Rev..

[3] Kumanotani J. (1995). Urushi (*Oriental lacquer*)—A natural aesthetic durable and future-promising coating. Prog. Org. Coat..

[4] Niimura N., Miyakoshi T. (2003). Characterization of natural resin films and identification of ancient coating. J. Mass Spectrom. Soc. Jpn..

[5] Snyder D.M. (1989). An overview of Oriental lacquer: Art and chemistry of the original high-tech coating. J. Chem. Educ..

[6] Harigaya S., Honda T., Rong L., Miyakoshi T., Chen C.-L. (2007). Enzymatic Dehydrogenative Polymerization of Urushiols in Fresh Exudates from the Lacquer Tree, Rhus Vernicifera DC. J. Agric. Food Chem..

[7] Brommelle N.S., Smith P. (1988). Urushi Proceedings of the Urushi Study Group.

[8] Vogl O. (2000). Oriental lacquer, poison ivy, and drying oils. J. Polym. Sci. A Polym. Chem..

[9] Suk K.T., Baik S.K., Kim H.S., Park S.M., Paeng K.J., Uh Y., Jang I.H., Cho M.Y., Choi E.H., Kim M.J. (2011). Anti-bacterial effects of the urushiol component in the sap of the lacquer tree (*Rhus verniciflua* Stokes) on Helicobacter pylori. Helicobacter.

[10] Jeong H., Heo J., Son B., Choi D., Park T.H., Chang M., Hong J. (2015). Intrinsic hydrophobic cairnlike multilayer films for antibacterial effect with enhanced durability. ACS Appl. Mater. Interfaces.

[11] Niimura N. (2009). Determination of the type of lacquer on East Asian lacquer ware. Int. J. Mass Spectrom..

[12] Terada T., Oda K., Oyabu H., Asami T. (1999). Urushi—The Science and Practice.

[13] Frade J.C., Ribeiro M.I., Graça J., Rodrigues J. (2009). Applying pyrolysis–gas chromatography/mass spectrometry to the identification of Oriental lacquers: Study of two lacquered shields. Anal. Bioanal. Chem..

[14] Tsukagoshi M., Kitahara Y., Takahashi S., Fujii T. (2012). Pyrolysis analysis of Japanese lacquer films: Direct probe-Li+ ion attachment mass spectrometry versus pyrolysis/gas chromatography/mass spectrometry. J. Anal. Appl. Pyrolysis.

[15] Hô A.L., Regert M., Marescot O., Duhamel C., Langlois J., Miyakoshi T., Genty C., Sablier M. (2012). Molecular criteria for discriminating museum Asian lacquerware from different vegetal origins by pyrolysis gas chromatography/mass spectrometry. Anal. Chim. Acta.

[16] Lee J., Jung S.-B., Terlier T., Lee K.-B., Lee Y. (2018). Molecular identification of Asian lacquers from different trees using Py–GC/MS and ToF–SIMS. Surf. Interface Anal..

[17] Yu H.H., Lim J.-A., Ham S.W., Lee K.-B., Lee Y. (2021). Quantitative analysis of blended Asian lacquers using ToF–SIMS, Py–GC/MS and HPLC. Polymers.

[18] Lu R., Hariygaya S., Ishimura T., Nagase K., Miyakoshi T. (2004). Development of a fast drying lacquer based on raw lacquer sap. Prog. Org. Coat..

[19] Wang C., Chen H., Zhou H., Li W., Lu L., Phuc B.T. (2014). Investigation and development on processing of Vietnamese lacquer. Adv. Biol. Chem..

[20] Niimura N. (2012). Structural study of a Japanese lacquer film with thermogravimetry-linked scan mass spectrometry. Int. J. Polym. Anal. Charact..

[21] Yang J., Chen N., Zhu J., Cai J., Deng J., Pan F., Gao L., Jiang Z., Shen F. (2020). Polymerization mechanism of natural lacquer sap with special phase structure. Sci. Rep..

[22] Sung M., Lu R., Miyakoshi T., Jung J. (2017). Thermal polymerization mechanism of thitsiol separated from Gluta usitata. Int. J. Polym. Anal. Charact..

[23] Niimura N., Iijima Y., Miyakoshi T. (1996). Hardening process and surface structure of lacquer films studied by X-ray photoelectron spectroscopy. Surf. Interface Anal..

[24] Park J.H., Park J.H., Kim S.C. (2020). A study on application of enzyme additives to improve drying speed of Urushi lacquer. J. Korean Wood Sci. Technol..

[25] Chang C.-W., Lee J.-J., Lu K.-T. (2020). The effects of adding different HALS on the curing process, film properties and light-fastness of refined Oriental lacquer. Polymers.

[26] Narita C., Okahisa Y., Yamada K. (2019). Plasticizing effect of lignin on urushi in bio-composite films. Polymer.

[27] Royall C.P., Donald A.M. (2002). Surface properties and structural collapse of silica in matte water-based lacquers. Langmuir.

[28] Tian Y., Guo K., Bian X., Wang T., Chen S., Sun J. (2017). Durable and room-temperature curable superhydrophobic composite coating on nitrocellulose lacquer. Surf. Coat. Technol..

[29] Xiao B., Zhang Y., Wang Y., Jiang G., Liang M., Chen X., Long G. (2019). A fractal model for kozeny–carman constant and dimensionless permeability of fibrous porous media with toughened surfaces. Fractals.

[30] Xiao B., Huang Q., Chen H., Chen X., Long G. (2021). A fractal model for capillary flow through a single tortuous capillary with roughened surfaces in fibrous porous media. Fractals.

[31] Chang C.-W., Lee H.-L., Lu K.-T. (2019). Manufacture and characteristics of oil-modified refined lacquer for wood coatings. Coatings.

[32] Honda T., Lu R., Sakai R., Ishimura T., Miyakoshi T. (2008). Characterization and comparison of Asian lacquer saps. Prog. Org. Coat..

[33] Anzai K., Lu R., Phuc B.T., Miyakoshi T. (2014). Development and characterization of laccol lacquer blended with urushiol lacquer. Int. J. Polym. Anal. Chem..

[34] Domene-López D., García-Quesada J.C., Martín-Gullón I. (2018). A correlation between the wolf-wilburn scale and atomic force microscopy for anti-scratch resistance determination. Prog. Org. Coat..

[35] Lee B.-H., Kim H.-J. (2004). Curing behaviors of *Korean Dendropanax* lacquer determined by chemical and physical measures. J. Appl. Polym. Sci..

[36] Lu R., Ishimura T., Tsutida K., Honda T., Miyakoshi T. (2005). Development of a fast drying hybrid lacquer in a low-relative-humidity environment based on kurome lacquer sap. J. Appl. Polym. Sci..

[37] EN ISO 2409: 2020 (2020). Paints and Varnishes—Cross-Cut Test.

[38] Oliver W.C., Pharr G.M. (2004). Measurement of hardness and elastic modulus by instrumented indentation: Advances in understanding and refinements to methodology. J. Mater. Res..

